# Assessment of genetic diversity in main local sheep breeds from Romania using microsatellite markers

**DOI:** 10.5194/aab-63-53-2020

**Published:** 2020-02-19

**Authors:** Andreea Dudu, Gina-Oana Popa, Elena Ghiță, Rodica Pelmuș, Cristina Lazăr, Marieta Costache, Sergiu E. Georgescu

**Affiliations:** 1 Department of Biochemistry and Molecular Biology, Faculty of Biology, University of Bucharest, Bucharest, 050095, Romania; 2 National Research and Development Institute for Animal Biology and Nutrition, Baloteşti, 077015, Romania

## Abstract

The state of the local breeds of farm animals is increasingly precarious worldwide because of the aggressive introduction of breeds with improved economical traits. The preference of the breeders for local breeds is due to their higher adaptability to the particular climate and relief conditions of the mountain areas, to the high rate of assimilation of the feeds from these regions and to their increased resistance to diseases. This study analyzes the genetic variation of the main four local Romanian sheep breeds (Tsurcana, Tsigai, Ratska and Teleorman Blackhead) in terms of stock and economic importance, using 18 microsatellite markers. The mean number of alleles per locus was of 9.764. The values of genetic diversity parameters exhibited a high degree of polymorphism for the analyzed breeds, although inbreeding was highlighted particularly in Tsurcana and Tsigai. These breeds also showed an intense gene flow among them and were less differentiated in comparison with Ratska and Teleorman Blackhead. The results of this study may be useful for breeding programs and conservation plans since the genetic resources of the local breeds must be preserved so as to maintain an adequate level of biodiversity in animal husbandry.

## 1 Introduction

The diversity of the local breeds in Romania is very high, even nowadays, firstly because of the high variety of relief forms, implicitly of the existing ecological systems, as well as because of the substantial inflow of animals from abroad, especially at the end of the First World War when territories of the former Austro-Hungarian Empire returned to Romania. However, significant erosion of the local genetic resources has been noticed as of the 20th century, but it seems that this phenomenon has affected the local Romanian sheep breeds rather little. This is due to the rearing of the different local breeds in limited, geographically isolated areas where the farmers use traditional systems.

In Romania there is an extremely broad variety of local sheep breeds. Tsurcana is the most numerous and widespread sheep breed from Romania and is the starting point of all Wallachian (Zackel) sheep breeds in central and eastern Europe (Ilişiu et al., 2013). This breed has good aptitudes for walking, as well as a high capacity for adapting to difficult environmental conditions, high resistance to diseases and a high capacity to use roughages. Ratska sheep have been long-time considered to be a variety of Tsurcana sheep, but now it is considered to be a different transboundary breed (Savic et al., 2013). In terms of the stock of sheep, this breed is in a good state of preservation in Hungary and in a critical state in Serbia; in Romania, it lost ground to other breeds, currently being raised only in a few locations in the Banat region, at the border with Serbia (Dudu et al., 2016). The Tsigai breed is thought to have originated in Asia Minor, and currently it is widespread in Ukraine, Czech Republic, Hungary and Serbia. In Romania, Tsigai sheep rank second in terms of stock of animals and area of rearing, being a dual-purpose breed, with good milk yields (Ilişiu et al., 2013). The Teleorman Blackhead is a local breed that has been reared for a long time in southern Romania, in the Danube meadows, which was homologated in 2010 under the name Teleorman Blackhead. This breed is very well adapted to meadows and plain areas, but it can successfully acclimatize to hill areas as well (Pelmus et al., 2012).

Many studies have been conducted in recent years on the genetic variability and diversity of the local sheep breeds, using microsatellites analysis. Some of these studies were done in the Balkans area, in Greece (Mastranestasis et al., 2015; Loukovitis et al., 2016), Turkey (Yilmaz et al., 2014), Bulgaria (Kusza et al., 2010) and Romania (Kevorkian et al., 2010). These studies reveal the rather precarious situation of these local breeds, which lose ground to the imported breeds, and the genetic diversity tends to decrease due to the shrinking numbers and because the stocks of sheep are reared increasingly isolated from one another.

The purpose of this study was to obtain information on the genetic diversity of the most important local sheep breeds from Romania, which are also widespread in central and eastern Europe, with the purpose of making an inventory of their genetic resources and of constructing a database which will be available for future programs of sustainable breeding and conservation.

## 2 Methods

### 2.1 Sampling and DNA extraction

A total of 308 blood samples were collected from four local sheep breeds: Tsurcana (78 samples), Ratska (82 samples), Teleorman Blackhead (72 samples) and Tsigai (76 samples). The samples come from unrelated animals reared by private producers living in different counties of Romania: Caraş-Severin County (Ratska), Teleorman County (Teleorman Blackhead), Cluj County (Tsigai), Argeș County (Tsurcana) and Dâmbovița County (Tsurcana and Tsigai). For sampling, three different flocks were selected from each breed. Blood samples were collected in compliance with the Directive 2010/63/EU of the European Parliament and of the Council of 22 September 2010 on the protection of animals used for scientific purposes, and all the efforts were made in order to minimize animal suffering. Also, no animals were affected in any way during the sampling.

The DNA was extracted with the Wizard Genomic DNA Purification Kit (Promega), and the quality and quantity were checked using a NanoDrop 8000 spectrophotometer (Thermo Scientific).

### 2.2 Genotyping

The 18 microsatellites (Table S1 in the Supplement) were amplified by three PCR multiplex reactions, as follows: 3-plex reaction for OarFCB11 (Buchanan and Crawford, 1993), MAF33 (Buchanan and Crawford, 1992a), and OarFCB20 (Buchanan et al., 1994); 8-plex reaction for OarCP20 (Buchanan et al., 1994), OarCP34 (Ede et al., 1995), MAF70 (Buchanan et al., 1993), MAF214 (Buchanan and Crawford, 1992b), MAF65 (Buchanan et al., 1992), BM143 (Maddox et al., 1996), McM42 (Dalvit et al., 2008) and HSC (Scott et al., 1991); 7-plex reaction for MAF35 (Swarbrick et al., 1991), OarCP49 (Ede et al., 1995), BM1314, TGLA53 (Bishop et al., 1994), INRA063, INRA127 (Vaiman et al., 1994), and McM527 (Hulme et al., 1994). The PCR reactions were done in a final volume of 25 µL containing polymerase buffer, 1.5 mM ${\mathrm{MgCl}}_{2}$, 200 µM dNTPs, 30 ng DNA template, 0.5 U of AmpliTaq Gold DNA Polymerase and nuclease-free water. The concentration of each primers pair was adjusted to ensure a rather equal amplification for each microsatellite. The amplification programs for PCR multiplex reactions were comprised of 35 cycles consisting in denaturation, for 30 s at 95 ${}^{\circ }$C, hybridization, for 30 s at 59 ${}^{\circ }$C, and extension, for 75 s at 72 ${}^{\circ }$C. The initial step was done at 95 ${}^{\circ }$C for 10 min, followed in the end by a step of extension for 60 min at 72 ${}^{\circ }$C. The PCR products were mixed and subjected to capillary electrophoresis with fluorescent detection using an ABIPrism310 Genetic Analyzer (Applied Biosystems). Gene-Scan 500 LIZ Size Standard (Applied Biosystems) was used as a molecular weight marker. The results were processed by GeneMapper 4.0 (Applied Biosystems).

**Table 1:** Genetic diversity parameters estimated for 18 microsatellite markers over all populations. TNA – total number of alleles; MNA – mean number of alleles; $N_{e}$ – effective number of alleles; $A_{r}$ – allelic richness; PIC – polymorphic information content for each locus; F statistics ($F_{\mathrm{is}}$, $F_{\mathrm{st}}$, $F_{\mathrm{it}}$); $H_{o}$ – observed heterozygosity; $H_{e}$ – expected heterozygosity; $H_{t}$ – Nei's gene diversity; $H_{s}$ – diversity within breeds; Dst – diversity between breeds; Gst – coefficient of gene differentiation; HWE – test for significant deviation from Hardy–Weinberg equilibrium with the hypothesis of the heterozygote excess (${}^{*}p\leq 0.05$; ${}^{**}p\leq 0.01$; ${}^{***}p\leq 0.001$).

Locus	TNA	MNA	$N_{e}$	$A_{r}$	PIC	$F_{\mathrm{is}}$	$F_{\mathrm{st}}$	$F_{\mathrm{it}}$	$H_{o}$	$H_{e}$	$H_{t}$	$H_{s}$	Dst	Gst	HWE
OarCP20	12	6.5	3.343	7.027	0.658	0.091	0.024	0.112	0.632	0.707	0.705	0.692	0.013	0.018	NS
OarCP34	9	6	4.056	6.544	0.754	0.083	0.047	0.126	0.697	0.788	0.786	0.757	0.029	0.037	NS
OarCP49	19	17.5	12.097	17.465	0.924	0.224	0.010	0.236	0.711	0.931	0.933	0.940	0.007	0.008	NS
MAF70	19	13	6.636	15.393	0.868	0.341	0.034	0.346	0.566	0.881	0.885	0.860	0.025	0.028	${}^{***}$
MAF65	13	9.25	4.360	9.771	0.754	0.121	0.015	0.135	0.684	0.788	0.788	0.779	0.010	0.012	NS
MAF33	15	8.5	4.791	10.867	0.819	0.182	0.070	0.239	0.651	0.841	0.838	0.793	0.045	0.053	NS
MAF35	9	9	7.157	8.859	0.859	0.138	0.018	0.146	0.750	0.876	0.875	0.870	0.016	0.018	NS
MAF214	11	6.25	3.063	8.125	0.668	0.227	0.036	0.255	0.526	0.700	0.703	0.683	0.020	0.029	${}^{*}$
BM143	11	8.5	4.825	9.717	0.801	0.223	0.030	0.246	0.625	0.823	0.822	0.803	0.019	0.023	NS
BM1314	17	10.25	8.014	15.914	0.925	0.213	0.088	0.283	0.684	0.932	0.931	0.864	0.067	0.072	NS
HSC	16	12.75	6.578	13.828	0.855	0.103	0.039	0.138	0.757	0.869	0.866	0.840	0.026	0.030	NS
McM42	12	7.5	3.517	8.882	0.731	0.054	0.060	0.111	0.689	0.763	0.761	0.725	0.035	0.047	NS
McM527	7	7	6.597	6.999	0.831	0.222	0.013	0.211	0.671	0.583	0.854	0.862	0.009	0.010	NS
OarFCB20	17	10.5	5.582	11.970	0.848	0.072	0.058	0.125	0.708	0.865	0.864	0.823	0.041	0.048	NS
OarFCB11	11	8.5	4.913	9.096	0.828	0.094	0.051	0.140	0.740	0.849	0.851	0.816	0.035	0.041	NS
INRA063	14	11.25	7.494	12.674	0.886	0.210	0.024	0.229	0.697	0.898	0.897	0.881	0.016	0.018	NS
INRA127	14	13.5	10.857	13.477	0.905	0.172	0.010	0.164	0.763	0.914	0.915	0.921	0.007	0.007	NS
TGLA53	12	10	6.985	11.845	0.896	0.064	0.062	0.121	0.809	0.907	0.908	0.865	0.043	0.048	NS
All loci	238	9.764	–	–	0.823	0.161	0.034	0.189	0.691	0.844	0.843	0.821	0.023	0.027	

### 2.3 Data analysis

Total number of alleles, allelic frequencies, total number of alleles per locus (TNA), mean number of alleles (MNA), effective number of alleles ($N_{e}$), observed heterozygosity ($H_{o}$) and expected heterozygosity ($H_{e}$) were calculated with GENETIX 4.05.2 (Belkhir et al., 2004) and GenAlEx 6.503 (Peakall and Smouse, 2012). Polymorphic information content (PIC) and Hardy–Weinberg equilibrium were calculated using CERVUS software. The estimates of Wright statistics indices per locus and overall loci and gene diversity, allelic richness per locus and population, Nei's gene diversity ($H_{t}$), diversity between breeds (Dst) and coefficient of gene differentiation (Gst) values and pairwise were calculated with FSTAT (Goudet, 1995). As a measure of the genetic distance between the breeds, we determined pairwise $F_{\mathrm{st}}$ for all pairs of populations using FSTAT software.

In order to infer the differentiation among the investigated breeds, we used a factorial correspondence analysis (FCA) implemented in Genetix 4.05.2. The genetic structure of the populations was analyzed using STRUCTURE software (Pritchard et al., 2000). The tests were performed using an admixture model, in which the allelic frequencies were correlated. In order to select the appropriate number of inferred populations, several analyses were conducted with K (number of populations inferred) ranging from 2 to 6, a total of 300 000 iterations (burn-in period 3000) and 10 independent replications for each analysis. The real K values were gathered using the Structure Harvester (Earl and Von Holdt, 2012), according to Evanno's method (Evanno et al., 2005). This algorithm offers the identification of the appropriate number of clusters using ΔK, based on the rate of change in the log probability of the data.

## 3 Results

### 3.1 Genetic variation among and within breeds

We tested all 18 loci with MICRO-CHECKER (Van Oosterhout et al., 2004) and did not detect evidence for genotype inferring errors due to stuttering, neither for large allele dropout nor for a high frequency of null alleles.

A total of 238 alleles were observed for the 18 analyzed loci. The characteristics of the analyzed loci along with the genetic variability statistics are summarized in Table 1. The total number of alleles per locus ranged from 9 (OarCP34) to 19 (MAF70), while the mean number of alleles per locus varied between 6 and 13 for the same loci, with a mean number of alleles per locus of 9.764. The effective number of alleles per locus ranged between 3.063 (MAF214) and 12.097 (OarCP49). The PIC values were between 0.658 (OarCP20) and 0.925 (BM1314), with a mean of 0.823 for all the loci.

Mean $H_{o}$ and $H_{e}$ were higher than 0.5 for all loci. However, the value of $H_{o}$ for all loci was lower than the value of $H_{e}$, indicating an excess of homozygosity. The values for $H_{e}$ ranged from 0.700 to 0.932, values that together with the ones of PIC demonstrate that the microsatellites were properly selected to infer the genetic variation (Takezaki and Nei, 1996). F statistics of overall loci were $F_{\mathrm{is}}=0.161$, $F_{\mathrm{it}}=0.189$ and $F_{\mathrm{st}}=0.034$. Mean $F_{\mathrm{st}}$ (0.034) was moderate to low while $H_{s}$ (0.821) was relatively high. Nei's gene diversity index ($H_{t}$) for loci ranged from 0.703 (MAF214) to 0.933 (OarCP49), with an average of 0.821 (Table 1). The $H_{o}$ values for Romanian breeds ranged from 0.652 for Tsurcana to 0.741 for Teleorman Blackhead (Table 2).

**Table 2:** Genetic variability for Romanian sheep breeds (standard error values are in the brackets). $N_{a}$ – number of different alleles; $N_{e}$ – number of effective alleles; MNA/locus – mean number of alleles per locus; $A_{r}$ – allelic richness; Par – private allelic richness; $H_{o}$ – observed heterozygosity; $H_{e}$ – expected heterozygosity; $F_{\mathrm{is}}$ – $F_{\mathrm{is}}$ values (averaged over all loci); Deviation HWE – deviations from Hardy–Weinberg equilibrium.

Population	$N_{a}$	$N_{e}$	MNA/locus	$A_{r}$	Par	$H_{o}$	$H_{e}$	$F_{\mathrm{is}}$	Deviation HWE
Ratska	8.444 (0.905)	5.549 (0.720)	8.1667	10.537	9	0.707 (0.022)	0.779 (0.021)	0.091	At locus 9, 14
Teleorman Blackhead	9.167 (0.746)	5.972 (0.625)	9.8889	9.994	7	0.741 (0.043)	0.801 (0.018)	0.141	–
Tsurcana	10.222 (0.790)	6.742 (0.750)	10.222	9.974	7	0.652 (0.026)	0.813 (0.019)	0.203	At locus 11
Tsigai	10.222 (1.054)	6.353 (0.637)	10.167	11.025	10	0.663 (0.029)	0.818 (0.020)	0.196	–

**Figure 1:**
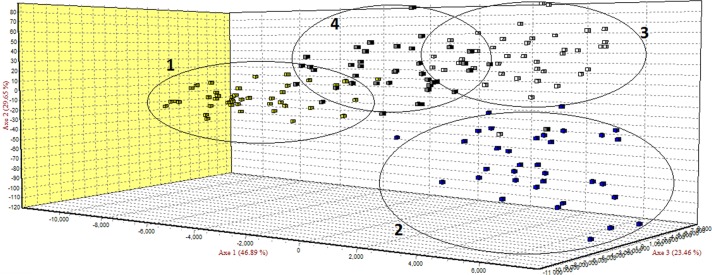
Factorial correspondence analysis (FCA) of four local sheep breeds from Romania: (1) Ratska, (2) Teleorman Blackhead, (3) Tsurcana and (4) Tsigai.

### 3.2 Genetic differentiation

The $F_{\mathrm{st}}$ values of pairwise comparisons among the Romanian sheep ranged from 0.02271 between Tsurcana and Tsigai to 0.08912 between Ratska and Teleorman Blackhead. The number of migrants ($N_{m}$) was correlated with the values of $F_{\mathrm{st}}$ and ranged from 10.76 between Tsurcana and Tsigai to 2.56 between Teleorman Blackhead and Ratska (Table 3).

**Table 3:** Pairwise genetic differentiation ($F_{\mathrm{st}}$) (above the diagonal) and number of migrants per generation ($N_{m}$) (below the diagonal).

	Ratska	Teleorman Blackhead	Tsurcana	Tsigai
Ratska	–	0.08912	0.07955	0.05527
Teleorman Blackhead	2.56	–	0.03722	0.03425
Tsurcana	2.89	6.47	–	0.02271
Tsigai	4.27	7.05	10.76	–

**Figure 2:**
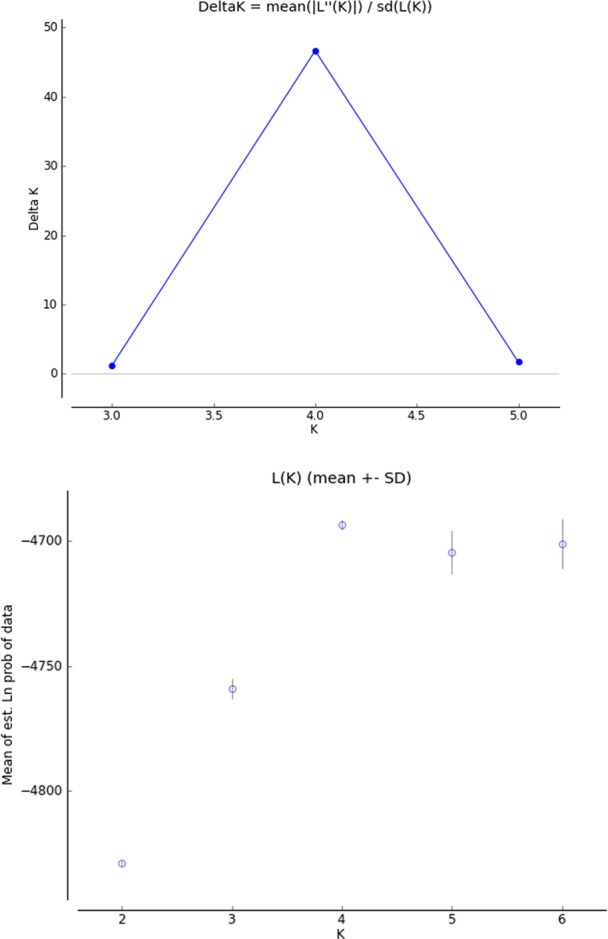
ΔK value inferred with Structure Harvester.

**Figure 3:**
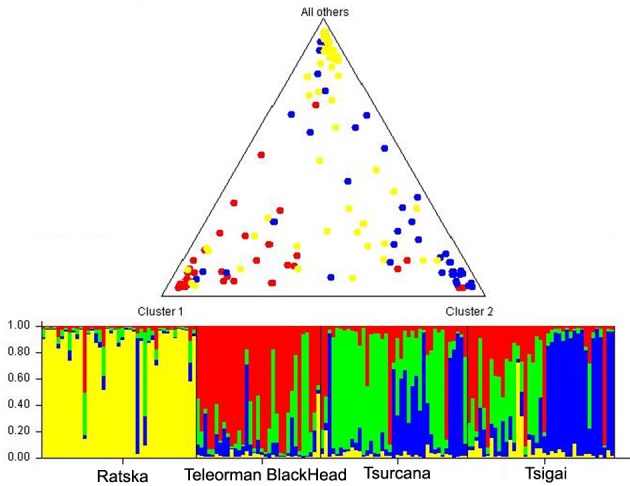
Genetic structure of Romanian breeds inferred by Bayesian analysis at K=4 using STRUCTURE program. Ratska – yellow; Teleorman Blackhead – red; Tsurcana – green; Tsigai – blue. The colored bar plots show the assignment of individual according to Q values. Triangle plot displays average admixture according of these four breeds.

The FCA analysis has shown that Ratska and Teleorman Blackhead are clearly separated, while between Tsurcana and Tsigai the separation is less noticeable (Fig. 1). According to the STRUCTURE analysis, the most likely value of ΔK was obtained for K=4, indicating that the four breeds analyzed in this study can be assigned to four clusters (Fig. 2). In graphical representation of the clustering breeds (Fig. 3), each color represents one cluster, and the length of the colored segment shows the individual's estimated proportion of membership in that cluster. Black lines separate the individuals of the four local Romanian breeds.

## 4 Discussion

The values of genetic diversity parameters were higher compared with similar study of Tsigai and Zackel type group sheep breeds from central, eastern and southern European regions (Kusza et al., 2008). Also, the mean $H_{e}$ value of all 18 loci (0.844) was higher than the values reported in the literature (Kusza et al., 2008, 2010, 2011; Neubauer et al., 2015). Positive values of $F_{\mathrm{is}}$ indicate loss of heterozygosity in all loci, similar with the results reported by Kusza et al. (2008, 2011), Kevorkian et al. (2010) and Zahan et al. (2011). The overall value of Dst (0.023) and the value of mean $F_{\mathrm{st}}$ (0.034) were low, indicating a low genetic diversity between breeds. The Gst value that shows the diversity within breeds relative to the diversity of the entire population is 0.027 and indicates that 2.7 % of total genetic variation is due to the differences between the populations. A total of 14 loci were in Hardy–Weinberg equilibrium, while MAF 214 and MAF70 deviate from this (Table 1).

Several indicators of variability within a breed like $N_{a}$, $N_{e}$ and MNA highlighted the highest values for Tsurcana and Tsigai, followed by Teleorman Blackhead and Ratska (Table 2). The values were higher than the ones reported in the literature for breeds from this region, such as Teleorman Blackhead and Tsigai (Kusza et al., 2008, 2011). The obtained $H_{e}$ values for all breeds were higher than $H_{o}$ values indicating that several factors, and mostly inbreeding, might contribute to less than expected heterozygosity in a population. The $F_{\mathrm{is}}$ values were positive but lower than the ones reported by Kusza et al. (2008, 2011). However, with the exception of the $F_{\mathrm{is}}$ value for Tsurcana, the rest are not significantly different from zero. The degree of inbreeding was higher in Tsurcana and Tsigai, followed by Ratska and Teleorman Blackhead. Regarding genetic differentiation, the highest degree of gene flow (highest $N_{m}$ value) was found between Tsurcana and Tsigai, which is also supported by the fact that the two breeds had the lowest $F_{\mathrm{st}}$ value among all pairwise comparisons (Table 3). This suggests that Tsurcana and Tsigai breeds might have a common history and breeding practices.

In the FCA analysis, Ratska and Teleorman Blackhead are clearly separated, while separation is less noticeable between Tsurcana and Tsigai. Teleorman Blackhead is grouped in a cluster differentiated from the rest of analyzed breeds, while Ratska and Tsurcana are also clearly separated and they have the second highest pairwise $F_{\mathrm{st}}$ value (Fig. 1). These findings are supported by the fact that Ratska and Teleorman Blackhead had the highest value of pairwise $F_{\mathrm{st}}$, while between Tsurcana and Tsigai there is a great gene flow. Overall, the differentiation patterns observed in the FCA analysis are generally in agreement with the pairwise $F_{\mathrm{st}}$ estimates of the studied breeds. According to the STRUCTURE analysis, Ratska and Teleorman Blackhead appear as two genetically distinct groups, while Tsurcana and Tsigai remain less differentiated.

## 5 Conclusions

The results showed high levels of genetic variability for all local sheep breeds from Romania. The $F_{\mathrm{is}}$ had positive values for all breeds, but they were significantly higher in Tsurcana and Tsigai, which also showed an intense gene flow between them and a low degree of genetic differentiation. Tsurcana and Tsigai breeds have a common history and mutual breeding practices with exchange of animals between flocks. This is also reflected in the clustering obtained by STRUCTURE analysis, which highlighted that Ratska and Teleorman Blackhead were well differentiated in comparison with Tsurcana and Tsigai. Overall, the level of genetic diversity could be attributed to lack of artificial selection pressure and high level of gene flow among breeds typical of traditional breeding systems.

## Supplement

10.5194/aab-63-53-2020-supplement The supplement related to this article is available online at: https://doi.org/10.5194/aab-63-53-2020-supplement.

## Data Availability

The data sets are available upon request from the corresponding author.
