# Supplementary material for: Assessment of genetic diversity in main local sheep breeds from Romania using microsatellite markers
Source: Arch Anim Breed. 2020 Feb 19;63(1):53–9. doi: 10.5194/aab-63-53-2020 (PMC7059605; doi:10.5194/aab-63-53-2020)
Supplement: The supplement related to this article is available online at: https://doi.org/10.5194/aab-63-53-2020-supplement. [file aab-63-53-supplement.pdf]

Supplement of Arch. Anim. Breed., 63, 53–59, 2020
https://doi.org/10.5194/aab-63-53-2020-supplement
© Author(s) 2020. This work is distributed under
the Creative Commons Attribution 4.0 License.

Open Access
Archives Animal Breeding

*Supplement of*

# Assessment of genetic diversity in main local sheep breeds from Romania using microsatellite markers

**Andreea Dudu et al.**

*Correspondence to:* Sergiu E. Georgescu (georgescu_se@yahoo.com, sergiu.georgescu@bio.unibuc.ro)

The copyright of individual parts of the supplement might differ from the CC BY 4.0 License.

**Table S1. The characteristics of the microsatellite markers used in the current study.**

| Locus | Primer sequence | GenBank accession number | Allelic range (bp) |
|---|---|---|---|
| OarCP20 | F: ggcatttcatggctttagcagg<br>R: gtttgatcccctggaggaggaaacgg | L01531 | 70-110 |
| OarCP34 | F: gctgaacaatgtgatatgttcagg<br>R: gggacaatactgtcttagatgctgc | U15699 | 102-120 |
| OarCP49 | F: cagacacggcttagcaactaaacgc<br>R: gtggggatgaatattccttcataagg | U15702 | 76-140 |
| MAF70 | F: cacggagtcacaaagagtcagacc<br>R: gcaggactctacggggcctttgc | M77199 | 124-160 |
| MAF65 | F: aaaggccagagtatgcaattaggag<br>R: ccactcctcctgagaatataacatg | M67437 | 120-144 |
| MAF33 | F: gatctttgtttcaatctattccaatttc<br>R: gatcatctgagtgtgagtatatacag | M77200 | 118-166 |
| MAF35 | F: agttacaaatgcaagcatcatacctg<br>R: tcaagaattttggagcacaattctgg | M80518 | 104-124 |
| MAF214 | F: aatgcaggagatctgaggcagggacg<br>R: gggtgatcttagggaggttttggagg | M88160 | 186-236 |
| BM143 | F:acctgggaagcctccatatc<br>R: ctgcaggcagattctttatcg | AF394448 | 98-118 |
| BM1314 | F: ttcctcctcttctctccaaac<br>R:atctcaaacgccagtgtgg | G18433 | 136-179 |
| HSC | F: ctgccaatgcagagacacaaga<br>R:gtctgtctcctgtcttgtcatc | M90759 | 265-295 |
| McM42 | F: catctttcaaaagaactccgaaagtg<br>R: cttggaatccttcctaactttcgg | L34281 | 73-103 |
| McM527 | F: gtccattgcctcaaatcaaattc<br>R: aaaccacttgactactccccaa | L34277 | 163-181 |
| OarFCB20 | F: ggaaaacccccatatatacctatac<br>R: aaatgtgtttaagattccatacatgtg | L20004 | 80-116 |
| OarFCB11 | F: gcaagcaggttctttaccactagcacc<br>R:ggcctgaactcacaagttgatatatctatcac | L01531 | 120-150 |
| INRA063 | F: atttgcacaagctaaatctaacc<br>R: aaaccacagaaatgcttggaag | X71507 | 155-211 |
| INRA127 | F:ctacagctctgatgagaacc<br>R: cgttttctcaaacttcattgcc | X71550 | 181-213 |
| TGLA53 | F: gctttcagaaatagtttgcattca<br>R: atcttcacatgatattacagcaga | - | 126-148 |
